# Supplementary material for: Active site specificity profiling datasets of matrix metalloproteinases (MMPs) 1, 2, 3, 7, 8, 9, 12, 13 and 14
Source: Data Brief. 2016 Feb 22;7:299–310. doi: 10.1016/j.dib.2016.02.036 (PMC4777984; doi:10.1016/j.dib.2016.02.036)
Supplement: Supplementary file 10 — Supplementary material [file mmc10.zip › WebPICS_hMMP1_G_1%/P2.html]

 

PICS results


|  |  |
| --- | --- |
| **P2\_A**  12 in 126 sites   9.5 %    effects > 10 perc. pnts.  (vice-versa in brackets)  P1prime\_C: 10.4 (15.5)   P2prime\_T: 25.4 (30.5)   P3prime\_T: 16.3 (17.8) |  |
| **P2\_C**  4 in 126 sites   3.2 %    effects > 10 perc. pnts.  (vice-versa in brackets)  P1\_H: 19.4 (11.1)   P2prime\_A: 40.5 (13.5)   P3prime\_K: 42.1 (16.8) |  |
| **P2\_F**  14 in 126 sites   11.1 %    effects > 10 perc. pnts.  (vice-versa in brackets)  P1\_N: 23.9 (13.9)   P3prime\_M: 11.1 (38.9)   P3prime\_N: 19.9 (25.3) |  |
| **P2\_K**  8 in 126 sites   6.3 %    effects > 10 perc. pnts.  (vice-versa in brackets)  P1\_A: 16.3 (11.9)   P2prime\_I: 15.5 (10.4) |  |
| **P2\_L**  16 in 126 sites   12.7 %    effects > 10 perc. pnts.  (vice-versa in brackets)  P3\_V: 12.3 (12.3)   P1\_P: 20.2 (54.0)   P2prime\_L: 12.3 (12.3)   P3prime\_K: 17.1 (27.3)   P3prime\_N: 10.1 (14.6)   P3prime\_Q: 10.1 (14.6) |  |
| **P2\_M**  6 in 126 sites   4.8 %    effects > 10 perc. pnts.  (vice-versa in brackets)  P2prime\_Y: 13.5 (20.2) |  |
| **P2\_N**  6 in 126 sites   4.8 %    effects > 10 perc. pnts.  (vice-versa in brackets)  P1\_A: 24.6 (13.4)   P1\_C: 12.7 (15.2)   P2prime\_T: 42.1 (25.2) |  |
| **P2\_Q**  14 in 126 sites   11.1 %    effects > 10 perc. pnts.  (vice-versa in brackets)  P1\_Y: 23.0 (46.0)   P2prime\_L: 23.0 (20.1)   P3prime\_Q: 19.9 (25.3) |  |
| **P2\_Y**  12 in 126 sites   9.5 %    effects > 10 perc. pnts.  (vice-versa in brackets)  P1\_S: 18.2 (11.6)   P1prime\_C: 10.4 (15.5)   P1prime\_I: 30.2 (14.5)   P2prime\_F: 20.2 (40.5)   P2prime\_V: 14.7 (13.6)   P2prime\_Y: 13.5 (40.5)   P3prime\_V: 20.6 (15.5) |  |
